# Long-Term Effectiveness of a Stress Management Intervention at Work: A 9-Year Follow-Up Study Based on a Randomized Wait-List Controlled Trial in Male Managers

**DOI:** 10.1155/2017/2853813

**Published:** 2017-10-18

**Authors:** Jian Li, Natalie Riedel, Amira Barrech, Raphael M. Herr, Birgit Aust, Kathrin Mörtl, Johannes Siegrist, Harald Gündel, Peter Angerer

**Affiliations:** ^1^Institute of Occupational, Social and Environmental Medicine, Centre for Health and Society, Faculty of Medicine, University of Düsseldorf, Düsseldorf, Germany; ^2^Department of Social Epidemiology, Institute of Public Health and Nursing Research, University of Bremen, Bremen, Germany; ^3^Department of Psychosomatic Medicine and Psychotherapy, Faculty of Medicine, University of Ulm, Ulm, Germany; ^4^Mannheim Institute of Public Health, Social and Preventive Medicine, Medical Faculty Mannheim, Heidelberg University, Mannheim, Germany; ^5^National Research Centre for the Working Environment, Copenhagen, Denmark; ^6^Department of Psychotherapy Science, Sigmund Freud Private University, Vienna, Austria; ^7^Life-Science Centre, University of Düsseldorf, Düsseldorf, Germany

## Abstract

**Objective:**

Short- and medium-term effectiveness (up to 3 years) of individual level stress management interventions (SMI) at work were demonstrated, yet long-term effectiveness remains unexplored. We therefore aimed to address this research gap.

**Methods:**

94 male middle managers participated in a randomized wait-list controlled trial between 2006 and 2008 and in a post-trial-follow-up survey in 2015. During the first two years, all received an 18-hour psychotherapeutic SMI intervention which was based on the Effort-Reward Imbalance (ERI) model: tackling stressor on mismatch between effort and reward and promoting recovery on overcommitment. Work stress (i.e., ERI indicators) was the primary outcome, and the secondary outcome was depressive symptoms. The long-term effectiveness of the SMI was examined by mixed modeling, using an external control group (*n* = 94).

**Results:**

Effort and reward were substantially improved with significant intervention ⁎ time interaction effects (*p* < 0.001) compared to the external control group; effects on overcommitment and depressive symptoms were also significant (*p* < 0.05 and *p* < 0.01, resp.), though their trajectories in the intervention group were less sustainable.

**Conclusions:**

The effectiveness of this psychotherapeutic SMI at work based on the ERI model was observed over a 9-year period, particularly on the effort-reward ratio.

## 1. Introduction

Chronic stress at work has been shown to be a risk factor for a range of diseases, including depression, cardiovascular disease, and musculoskeletal disorders [1]. Moreover, the societal costs of work stress are high. According to a recent report from the European Agency for Safety and Health at Work [2], it was estimated that the annual economic burden of work stress amounts to approximately EUR 617 billion in Europe and EUR 219 billion in the US. Consequently, work stress interventions provide a promising approach towards reducing stress levels in the workplace [3]. In theory, it has been suggested that workplace interventions at the organizational level targeting work conditions would exert powerful effects [4, 5]. In practice, however, organizational interventions are less frequent because of their high complexity and costs [4]. Therefore, individual level stress management interventions (SMIs) in the workplace have received increasing attention by researchers as well as by employers and employees [6, 7].

As evident from large surveillance data, the trends of work stress have recently been aggravated, mostly so in the context of economic globalization. The major current psychosocial stressors in the workplace are work intensification, job insecurity, low pay, and lack of opportunity [8, 9]. This phenomenon is well captured by an established work stress model, Effort-Reward Imbalance (ERI) [10]. The situation-specific component of the ERI model emphasizes the harmful effects of failed reciprocity between efforts spent at work and rewards received in turn (high effort/low reward), where rewards include salary, promotion prospects, esteem, and job security. In addition, the person-specific component of the ERI model termed overcommitment (OC) identifies a distinct pattern of coping with demanding situations characterized by an inability to withdraw from work obligations. Both components of this model contribute to sustained experience of stress, triggering psychoneuroendocrine arousal of the organism with adverse long-term effects on health [11].

In the past years, a number of individual level SMIs at work based on the ERI model have been conducted, and they reported positive effects on work stress and mental health [12–15]. However, when asking the critical question “how long do effects of an individual level SMI in the workplace last?” no robust answer is available as current knowledge concerning important outcomes such as work stress or mental health is restricted to short-term evaluations, usually weeks or months only [6], or, in one study, up to 3 years [16]. In general, worksite SMIs at the individual level with the largest effects on mental wellbeing are based on psychotherapeutic techniques, usually based on cognitive behavioural therapy (CBT) [6, 7, 17] and, more recently, on psychodynamic therapy [15]. In clinical settings, both therapies have been proven to be efficacious in a wide range of common mental disorders [18, 19]. Recent evidence demonstrated sustained improvement for depression after psychodynamic psychotherapy in terms of symptoms, work ability, personality, and social functioning [20] over a period up to ten years, even after short-term treatment. Similar effects have been demonstrated for CBT [21]. The long-term effectiveness of different types of psychotherapy on depression has been confirmed by a recent meta-analysis [22]. Thus, we assume that an individual level SMI, comparable to a short-term (12 × 90 minutes) group psychotherapy that draws on psychotherapeutic techniques, might have notable long-term effects on psychosocial stress levels among workers, if applied in an appropriate occupational setting. An individual level SMI in the workplace was conducted by our research team, starting in 2006 (see [15]). This randomized controlled trial with a 1-year follow-up period showed the SMI to be effective in improving stress management abilities (significant reduction of perceived stress reactivity). The trial also demonstrated a tendency towards work stress reduction and mental health improvement. Importantly, this study was the first one to apply psychodynamic principles in combination with cognitive behavioural techniques. Moreover, it was explicitly based on the ERI model, addressing both situation-specific and person-specific components.

Recently, in 2015, we conducted a post-trial-follow-up survey to explore the long-term effectiveness of this SMI at work. Preliminary results suggest that some positive effects on work stress and mental health are based on differences in these measures between three time points: preintervention in 2006, postintervention in 2008, and post-trial-follow-up in 2015. Because the initial wait-list control group also received the SMI, the findings provided evidence on effectiveness in the internal before-after comparisons only, without providing findings of a respective control group [23]. Therefore, we decided to recruit a post hoc external control group with corresponding sociodemographic characteristics, providing comparable longitudinal data on work stress and mental health. It is the aim of this current report to examine the long-term effectiveness on work stress and mental health of our workplace SMI at individual level, by incorporating data from this external control group.

## 2. Materials and Methods

### 2.1. Participants and Procedures

**Figure 1:**
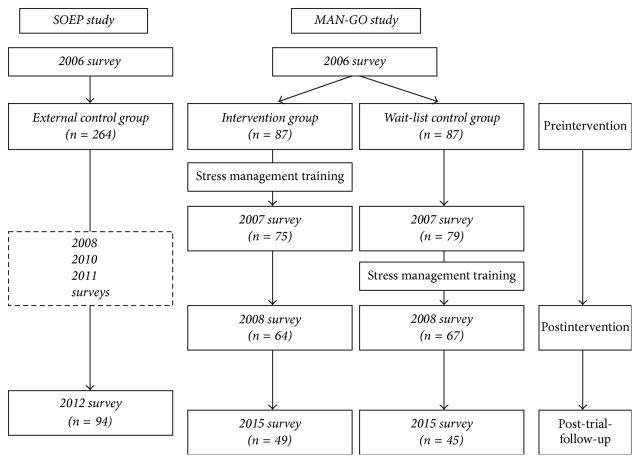
Flow-chart of participants of MAN-GO study and SOEP study.

The details of the randomized controlled trial were described elsewhere [15]. In summary, this SMI at work (MAN-GO study) was conducted in an international manufacturing plant located in Southern Germany. All lower and middle level managers from the blue collar sector who were responsible for a specific unit within production and for the management of on average 50 workers were eligible (*n* = 262). Typically, they were in a stressful “sandwich” position between higher management (engineers, business economists) and production. The inclusion criteria of the study were (1) male lower or middle level manager in the production department with leadership responsibility and (2) to be 18–65 years old with more than two years left before retirement. In 2006, 189 out of 262 subjects agreed to participate in this study, 15 subjects were further excluded due to not meeting the inclusion criteria, and finally 174 participants were randomized after the initial evaluation into the intervention group or the wait-list control group. The intervention group was offered a SMI in 2007, while the wait-list control group also received the same SMI in 2008. Surveys were conducted at baseline (2006) and after the SMI training in 2007 (*n* = 154) and 2008 (*n* = 131), respectively. For the current study, we conducted a post-trial-follow-up survey in 2015. In total, 94 participants who completed all questionnaire surveys in 2006, 2007, 2008, and 2015 were included for current analyses (Figure 1).

Given the fact that both the intervention group and the wait-list control group received the SMI, the two groups were merged for the current analysis and termed “MAN-GO participants.” In order to evaluate the long-term effectiveness of this SMI at work, an “unexposed” external control group was established post hoc by using data from the German Socioeconomic Panel (SOEP), offering a sample with comparable sociodemographic characteristics. SOEP is a well-established cohort study initiated in 1984 with annual survey waves, covering a large and representative sample of the German adult population [24]. Recently it has been recommended to use the SOEP data base as reference for comparison purposes [25]. Accordingly, we identified 264 eligible participants from the SOEP study in 2006, using the same inclusion criteria as the MAN-GO study (i.e., (1) male lower or middle level manager in the production department with leadership responsibility and (2) being 18–65 years old with more than two years left before retirement). In the SOEP study, work stress in terms of ERI was repeatedly measured in 2006 and 2011, while mental health (depressive symptoms) was repeatedly measured in 2006, 2008, 2010, and 2012. Since we needed participants who had participated in all of these surveys, the subjects who were lost during the follow-up were excluded. Therefore, the final external control group consisted of 94 SOEP participants (Figure 1).

This study was approved by the Ethical Committee of the University of Ulm and was performed in accordance with the ethical standards of the relevant national and institutional committees on human experimentation laid down in the 1975 Declaration of Helsinki and its later amendments. Written informed consent was obtained from each participant.

### 2.2. Intervention

A specifically tailored group-oriented stress intervention seminar (eight teaching units lasting 90 minutes each, spread over two consecutive days) was conducted in groups of 8–10 participants. In addition to a basic psychodynamic-oriented approach, elements of CBT were added in order to create an intervention which is best suitable for the mainly male participants. As for CBT elements, we especially used psychoeducation, working on cognitions, individual goal setting [26] if appropriate, and most importantly enhancing individual resources as well as resources within the whole group. As for psychodynamic therapeutic methods, we initially (or in the middle of the workshop) focused especially on negative, stressful emotions with an origin in the workplace setting, that is, intra- and interpersonal conflict involving intense emotions, and only when necessary sometimes addressed interpersonal conflicts, often with supervisors or colleagues, on the background of one's own biography. Overall, we used principles of modern psychodynamic group therapy enriched with CBT elements [27]. The work stress management was explicitly performed according to the parts of the ERI theory [10]: (1) stressor identifying and coping intervention for the situation-specific component of the ERI model (mismatch between effort and reward) and (2) recovery intervention for the personality-specific component of the ERI model if applicable (overcommitment). The seminar was provided by two experienced trainers, one in psychotherapy and the other one in occupational medicine. As one core element of applied psychotherapeutic techniques participants were asked to recall significant individual situations of stress in the workplace, identified by questions like “What were the most severe topics within work during the last months that really had some sustained negative impact on you, i.e., repetitively disturbing your sleep or coming to your mind during the night?” Once remembered, the individually significant conflictual situations were shared with another group member who then gave his personal assessment of the stressful situation, and how he would have handled it (“empathy exercise”). After the pairwise exercise, the individual stressful situations were reported to the entire group, one after the other, during the following sessions, and discussed to enhance comprehension of what specifically made them stressful. With the help of the seminar trainers, the group supported the stressed person to find his individually best possible solutions. During this group process, several “tools” to cope with difficult and stressful situations were introduced by the trainers, that is, how to deal with negative emotions such as sudden anger, how to handle and control individual impulsivity, or, more general, how to cope more effectively with interpersonal conflicts, how to improve social competence, how to reduce feelings of isolation by creating a social network, and how to detach from stressful events and thus recover from work [13, 15]. Additionally, the seminar was followed by two refresher courses (booster sessions) within 3–6 months, comprising two lessons/teaching units each (180 min per session).

### 2.3. Measures

Sociodemographic information was collected in 2006 before the start of the intervention, including age, education, and marital status. These have been identified as main risk factors of depression according to the US Preventive Services Task Force Recommendation Statement regarding the screening for depression in adults [28] and also represent critical covariates in work stress research, especially in ERI research [29]. In this study, the primary outcome was work stress based on the ERI theory, and the secondary outcome was depressive symptoms for mental health.

The standard short ERI questionnaire was applied which consists of three scales, effort (3 items), reward (7 items), and overcommitment (6 items) [29]. Responses to the items of effort and reward are scored on a 5-point scale where a value of “1” indicates no respective stressful experience and a value of “5” indicates very high stressful experience. Items of the scale overcommitment are scored on a 4-point scale (1 = full disagreement, 4 = full agreement with statement). Consequently, with such a scoring, the range for the scale effort is 3–15, for the scale reward 7–35, and for the scale overcommitment 6–24 with higher scores reflecting higher effort, reward, and overcommitment, respectively. According to a predefined algorithm, a ratio between the two scales effort and reward (weighted by item numbers) was calculated to quantify the degree of mismatch between high cost and low gain at work at individual level [29]. In the MAN-GO participants work stress was measured in 2006, 2007, 2008, and 2015, while it was only measured in 2006 and 2011 in the SOEP participants. In general, Cronbach's alpha coefficients of the ERI questionnaires in the MAN-GO study and the SOEP study were satisfactory (effort scale ranged from 0.67 to 0.80, reward scale ranged from 0.76 to 0.86, and overcommitment scale ranged from 0.72 to 0.81).

In the MAN-GO participants, depressive symptoms were measured by 7 items derived from the Hospital Anxiety and Depression Scale (HADS) [30]. The answer format of the HADS has four degrees coded from 0 to 3, resulting in a range of depressive symptoms from 0 to 21. Mental health in the SOEP participants was measured by the Short-Form 12 health survey (SF-12). Recently the mental component of the SF-12 (ranging from 0 to 100) has been approved as a measure of depressive symptoms [31] and assessed to be comparable to other standard depression measures including HADS (bivariate correlation between them was 0.70, based on pooled data from Germany) [32]. In order to be comparable to the SF-12 score (0–100), the original score of HADS-depression (0–21) was transformed to *Z*-score based on the normative values in Germany [33] and then reranged to 0–100, following the standardized “norm-based scoring” procedure of computing the SF-12 scores in Germany [34]. Several studies have indicated good sensitivity to changes over time for the HADS [35, 36], as well as SF-12 [37, 38]. In our current study for both MAN-GO participants and SOEP participants, the range of depressive symptoms was 0–100, where a high value reflected severe depressive mood. The HADS was repeatedly measured in 2006, 2007, 2008, and 2015 in the MAN-GO participants with satisfactory reliability (Cronbach's alpha coefficients ranged from 0.73 to 0.86), while the SF-12 was repeatedly measured in 2006, 2008, 2010, and 2012 in the SOEP participants with good reliability (Cronbach's alpha coefficients ranged from 0.88 to 0.91). Among the MAN-GO participants, both HADS and SF-12 were used in 2015. The correlation coefficient between HADS-depressive symptoms and SF-12-depressive symptoms was 0.80, providing further confirmation of the comparability between HADS and SF-12.

### 2.4. Statistical Analysis

The pooled sample (including the intervention group and the wait-list control group who all received the same intervention training) was compared with an external control group selected from an observational cohort study based on the same inclusion criteria. This approach involves some methodological challenges. Specifically, given the different time points of data collection between the MAN-GO and the SOEP studies, the unequal spacing of time intervals in repeated measurement (subjects are each observed at different sets of times and missing data are often unavoidable) and the correlated responses (the set of observations on one subject tends to be correlated) need to be addressed. Applying mixed regression modeling has been proposed to this end as an appropriate statistical approach to handle longitudinal data with repeated measures, and this approach has been often applied in research of intervention trials [39]. Therefore, we used mixed regression to examine the longitudinal tracking of changes in work stress and depressive symptoms during 2006–2015 between the MAN-GO participants and the SOEP participants. In our statistical modeling, “year” was centered at the middle time point of the study period (i.e., between 2006 and 2015) to reduce multicollinearity. Moreover, a year-squared term was included in order to deal with possible nonlinear relation of “year” with “work stress” or “depressive symptoms.” The intervention effect, the time effect, and the intervention *∗* time interaction effect were all tested. We performed data analyses with the statistical software SAS 9.4 (particularly SAS procedure PROC MIXED with default maximum likelihood estimation).

## 3. Results

### 3.1. Characteristics of Study Participants

Among the 94 male MAN-GO participants at baseline in 2006, the mean age was 40.6 years, most of them lived with their partners, and more than half had a low educational attainment (no more than 9 formal years); the scores of *E*-*R* ratio, overcommitment, and depressive symptoms were 0.74, 13.98, and 48.66, respectively. No differences in the sociodemographic characteristics, levels of work stress, and depressive symptoms were detected between MAN-GO participants and SOEP participants, indicating comparability of the two groups (Table 1).

### 3.2. Long-Term Effectiveness

**Figure 2:**
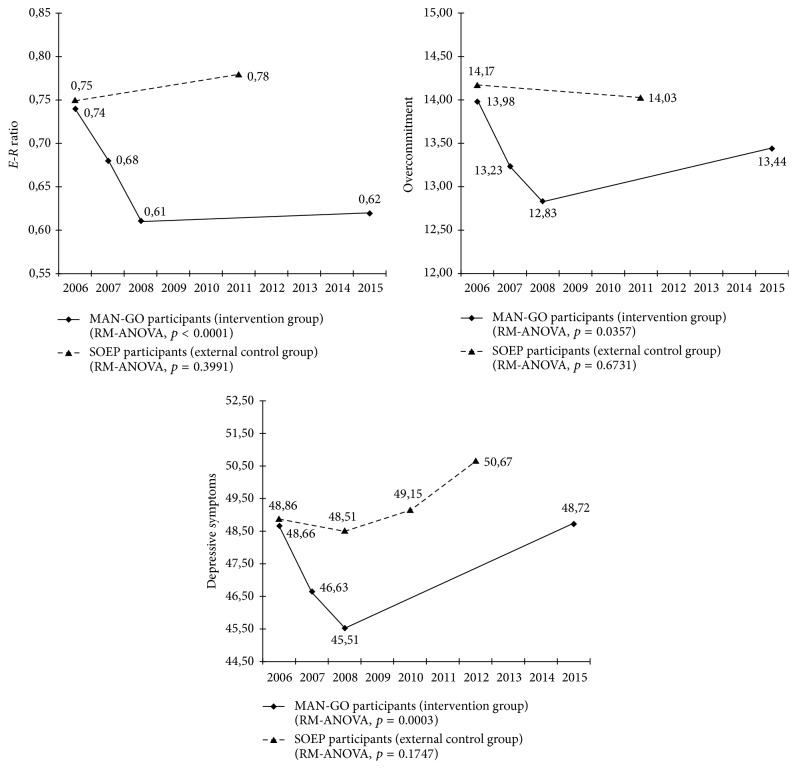
Development of work stress and depressive symptoms during 2006–2015 among MAN-GO participants (*n* = 94) and SOEP participants (*n* = 94). RM-ANOVA: repeated measures analysis of variance.

As displayed in Figure 2, in the MAN-GO participants, the ratio between effort and reward sharply decreased during the intervention period (2006–2008), and they remained relatively unchanged during the post-trial-follow-up (2008–2015). The pattern of overcommitment and depressive symptoms was somewhat different. Here, we observe a sharp decrease during the intervention period, but a steady increase during the post-trial-follow-up, nearly close to the level at preintervention (for overcommitment) or slightly beyond the level at preintervention (for depressive symptoms). With respect to the external control group, the longitudinal tracking of work stress and depressive symptoms in the SOEP participants clearly differed from the one observed in the MAN-GO participants: the *E*-*R* ratio slightly increased over time, whereas the mean score of overcommitment slightly decreased. To some extent, depressive symptoms elevated over time. We therefore additionally conducted repeated measures analysis of variance (RM-ANOVA) to examine the within-group changes of all work stress indicators and depressive symptoms across time points among MAN-GO participants and SOEP participants, respectively. Interestingly, we found all the variables demonstrated significant changes across 2006–2015 among MAN-GO participants (*p* < 0.05), whereas the changes among the SOEP participants were not significant (*p* > 0.05) (Table 2).

Table 3 shows the results of mixed modeling testing the long-term effectiveness between the intervention group and external control group. The nonlinear relations between “year” and “work stress” or “depressive symptoms” were taken in account, as a year-squared term was included (quadratic effects of “year” were significant *p* < 0.05 in all mixed regression models, data not shown). Compared to the external SOEP participants, the *E*-*R* ratio in the MAN-GO participants was substantially improved (effort was reduced and reward was increased), a significant intervention *∗* time interaction term was observed (*p* < 0.001), and overcommitment was clearly reduced, with a significant intervention *∗* time effect (*p* < 0.05). Regarding the secondary outcome, depressive symptoms, a similar longitudinal change was found, where depressive symptoms were significantly decreased (*p* < 0.01 on intervention *∗* time effect) in the MAN-GO participants. Additional adjustment for age, partnership, and education at baseline (preintervention in 2006) did not substantially attenuate or strengthen the magnitude of the reported effects (Table 4), further confirming the comparability of sociodemographic characteristics between MAN-GO participants and SOEP participants.

## 4. Discussion

Our 9-year follow-up study based on a randomized wait-list controlled trial found that this SMI in the workplace according to a well-established work stress model (i.e., ERI theory), in particular, using psychodynamic and cognitive behavioural techniques, showed significant long-term effectiveness preliminarily (for details, please see [23]) and extensively (this current report, with post hoc external control group), particularly on the effort-reward ratio (*p* < 0.001); though the trajectory of overcommitment and depressive symptoms did not sustain to the same extent, the long-term effects on them remained significant (*p* < 0.05 and *p* < 0.01, resp.). To the best of our knowledge, three ERI-based work stress intervention studies at individual level have been conducted. Mino and colleagues [12] reported a 3-month randomized controlled trial from Japan with significant effects on depression. Another two randomized controlled trials from Germany found the effects of SMI on work stress and burnout could last for 6 months [13] or 1 year [14, 40]. Additionally, our current study adds a new piece of scientific evidence, for the first time, that an individual level SMI in the workplace could produce long-term effectiveness over a time course of some 9 years, given the fact that previous worksite SMIs reported short- or medium-term effects (no longer than 3 years) only [6, 16].

Several features of our study might contribute to an explanation of why our worksite SMI can maintain such long-term effectiveness. First, one earlier intervention study based on the ERI model found that people who were highly motivated to participate benefited most [41]. Our MAN-GO study actively involved members of the workers' association (“Meisterverein”) representing our target group in the planning of the study. They iteratively contributed to the intervention design and the organization of the project. This early participation may have contributed to the high proportion of the target group (participation rate 72% [for details see [15]]) that took part, and may have had some additional influence as most participating workers had a genuinely high desire to reduce their stress levels at work. Thus, the procedure was in line with the principle of participatory action research which represents a particularly promising strategy [42]. Second, concerning the nature of our intervention, both cognitive behavioural and psychodynamic techniques were used. Whereas the CBT is commonly used in work stress intervention studies [6, 7, 17], the latter one have not been implemented previously in this context (see [15]). Referring to the long-term effects of psychodynamic therapy on depression [20], it may well be that this specific combination of intervention principles exerted sustained beneficial effects on participants as they were especially motivated to focus on and express their emotions and to interpret their health complaints in the daily context of psychosocial stress experience, specifically so at work. Psychotherapy research has also shown that studies providing booster sessions are likely to contribute to better treatment results compared to studies that do not provide any further support [22]. Moreover, social support experienced within the intervention setting may have contributed to a long-term maintenance of participants' improved coping behaviour. Thus, this study has extended research findings of long-term effectiveness of psychotherapy on common mental disorders from clinical settings to occupational settings as far as stress management is concerned. Third, most previous ERI-based intervention studies focused on its situation-specific components [12–14, 40], while one study only examined the short-term effectiveness of intervention on the person-specific component of overcommitment [13]. Our study included both components by identifying and addressing situation-specific aspects as well as by modifying critical individual behaviour (overcommitment). In recent years, intervention research examined the effects of improved coping with stressful work in more detail. For instance, several studies reported beneficial effects of becoming psychologically detached from work on workers' wellbeing [43, 44]. The notion of “psychological detachment from work” bears some resemblance with the concept of overcommitment applied in our study as the ability to withdraw from work obligations is a key aim of reducing people's excessive striving [10, 13]. Therefore, it is likely that an improved ability to recover from the strain caused by work stress may have contributed to the observed beneficial long-term effects. Finally, although our worksite SMI was focused at the individual level, one of the study aims was “in the long run, empowering the individual to influence workplace conditions” [15, page 127]. This aim can partially be achieved by strengthening the skills and competences that participants had acquired through our stress intervention seminar, empowering them to propose and negotiate distinct improvements of their work organization. These improvements may concern a more equitable distribution of work obligations, improved opportunities of training and promotion, the development of a culture of appreciation and recognition within the organization, and an implementation of material and nonmaterial incentives to reduce the imbalance between high effort and low reward at work. Clearly, measures at the organizational level need to be supplemented by labor and social policies at the national level in order to produce long-term sustainable effects [45]. It should be pointed out that, since the end of 2013, the Safety and Health at Work Act, which is a national law in Germany, explicitly requires every company to conduct regular psychosocial risk assessment among employees [46]. This significant change regarding work stress management at the national level might also help to reinforce the impact of interventions like our study on working people's health and wellbeing.

This current report suffers from several limitations. First, due to the original RCT design applying a wait-list control group, no internal control group was available at the time of the follow-up survey. Introducing an external control group as a post hoc comparison group must be considered critically when concerning research methodology, because the power of evaluating long-term effectiveness would be certainly reduced. As this group could not be randomized we cannot rule out that unmeasured differences may introduce a systematic bias, such as motivation to participate in the study and desire to change their working conditions. In addition, the MAN-GO participants were from a company, whereas the participants of the post hoc external control group were derived from a national cohort. Accordingly the findings based on mixed statistical approach should be interpreted cautiously, because clustering was considered within subjects (years) not between subjects (e.g., cluster of companies). However, there were no statistically significant differences in sociodemographic variables, job title, or outcome variables at baseline between the intervention group and the post hoc external control group. Second, due to the different measures of depressive symptoms (HADS in the intervention group and SF-12 in the external control group), a firm conclusion concerning the effect on depressive symptoms may not be feasible. In particular, depressive symptoms among the MAN-GO group were sharply decreased during the intervention period, but it was climbed up to the level at preintervention during the post-trial-follow-up period. As the secondary outcome of our study, mental health might be improved shortly after the reduction of work stress. However, multiple factors contribute to the trajectory of depressive symptoms. For example, the impact of macrosocial context should be considered, that is, the impact of Euro crisis which started from 2008 when our SMI at work was just completed [47, 48]. It is plausible the Euro crisis offsets the effect of intervention on mental health. Third, due to a high portion of men working in this part of the industry, no women were included in this study. Moreover, given the restriction to men with middle level leadership position, the generalization of the study findings is clearly limited [49]. Prior ERI-based SMIs at work which included both men and women [14, 40] did not identify obvious gender differences regarding the effect size, suggesting that our findings could also be applicable to employed women. Fourth, among 262 eligible subjects, 73 (28%) refused to participate in the initial MAN-GO study. The reason was not clear, the nonparticipants might have low motivation, or they were not able to join the intervention activities due to health/work/family issues. Moreover, the considerable attrition rates (i.e., sample reduction in the intervention group from 174 to 94 and in the post hoc external control group from 264 to 94) during the follow-up deserved attention. This loss might also relate to a healthy worker effect, such that some workers might have been unable to participate in the follow-up surveys covering a 9-year period, either due to severe health problems or due to employment-related organizational changes. Additional drop-out analyses were conducted to check whether the two groups of initial participants and those who remained till study end differed in main characteristics; we nevertheless did not observe any significant differences between those who continued to participate in surveys and those who dropped out during the follow-up (for details, please see the Supplementary Tables 1 and 2 available online at https://doi.org/10.1155/2017/2853813).

Finally, we need to bear in mind that the SMI of our study was mainly focused at the individual level. In general, individual interventions do not primarily target sources of stress at work. They are therefore limited in terms of their impact on some organizational outcomes [50]. It has been argued that organizational level workplace interventions will produce more sustainable effects on the health of employees than interventions targeting the individual level [4, 5]. In spite of some successful evidence of organizational interventions [e.g., [51–53]], the findings of organizational interventions are not consistent. These inconsistencies may result from differences in the implementation process and methodological difficulties in the evaluation [54–57]. Also, according to recent literature reviews [3, 6, 7], SMIs in the workplace combining both organizational and individual levels may be more effective. Notably, two large quasi-experimental studies at the organizational level from Canada provided evidence of medium-term effectiveness of work stress intervention by producing a decline of work stress in terms of the *E*-*R* ratio, as well as an improvement of mental and physical health in terms of reduced burnout and reduced musculoskeletal disorders [58, 59]. Thus, our intervention may serve as a starting point for future, more comprehensive approaches combining the individual and organizational levels.

## 5. Conclusions

In conclusion, this 9-year follow-up study based on a randomized wait-list controlled trial provides preliminary evidence of the long-term effectiveness of a SMI in the workplace, particularly on the effort-reward ratio, while long-term effects on overcommitment and depressive symptoms were less pronounced. An intervention based on psychodynamic and cognitive behavioural techniques and rooted in a solid stress theoretical framework, such as the ERI model, seems to be a promising tool for work stress management.

## Supplementary Material

Supplementary Table 1 is for drop out analysis among the MAN-GO participants. Supplementary Table 2 is for drop out analysis among the SOEP participants.

## Figures and Tables

**Table 1:** Comparison of sociodemographic characteristics as well as work stress and depressive symptoms measured at preintervention (2006) between MAN-GO participants (*n* = 94) and SOEP participants (*n* = 94).

Variable	MAN-GO participants	SOEP participants	*p* for difference
*Sociodemography*			
Mean age	40.60 ± 6.58	41.60 ± 7.44	0.33
Partner (versus no partner)	86 (91.49%)	78 (82.98%)	0.08
Low education (versus medium or high)	55 (58.51%)	52 (55.32%)	0.66
*Work stress*			
*E*-*R* ratio	0.74 ± 0.23	0.75 ± 0.40	0.81
(i) Effort	8.67 ± 1.78	8.39 ± 3.12	0.46
(ii) Reward	28.65 ± 4.93	28.62 ± 5.74	0.97
Overcommitment	13.98 ± 3.64	14.17 ± 3.21	0.71
*Mental health*			
Depressive symptoms	48.66 ± 8.21	48.86 ± 7.82	0.87

Means and standard deviations (SDs) for continuous variables and absolute numbers and per cent for categorical variables. Differences were examined by *t*-test for continuous variables and Chi-square test for categorical variables.

**Table 2:** Work stress and depressive symptoms across 2006–2015 among MAN-GO participants (*n* = 94) and SOEP participants (*n* = 94).

	2006	2007	2008	2010	2011	2012	2015	*p* for difference
				MAN-GO participants				
*Work stress*								
*E*-*R* ratio	0.74 ± 0.23	0.68 ± 0.26	0.61 ± 0.28				0.62 ± 0.25	<0.0001
(i) Effort	8.67 ± 1.78	8.23 ± 2.05	7.71 ± 2.40				7.91 ± 2.49	0.0004
(ii) Reward	28.65 ± 4.93	29.65 ± 4.74	31.18 ± 4.71				30.85 ± 4.81	<0.0001
Overcommitment	13.98 ± 3.64	13.23 ± 3.60	12.83 ± 3.42				13.44 ± 3.92	0.0357
*Mental health*								
Depressive symptoms	48.66 ± 8.21	46.63 ± 7.30	45.51 ± 7.23				48.72 ± 9.89	0.0003

				SOEP participants				
*Work stress*								
*E*-*R* ratio	0.75 ± 0.40				0.78 ± 0.47			0.3991
(i) Effort	8.39 ± 3.12				8.58 ± 3.00			0.5677
(ii) Reward	28.62 ± 5.74				28.65 ± 6.19			0.9485
Overcommitment	14.17 ± 3.21				14.03 ± 3.27			0.6731
*Mental health*								
Depressive symptoms	48.86 ± 7.82		48.51 ± 7.47	49.15 ± 9.16		50.67 ± 7.97		0.1747

Means and SDs; repeated measures analysis of variance.

**Table 3:** Longitudinal tracking of changes in work stress and depressive symptoms across 2006–2015 between MAN-GO participants (*n* = 94) and SOEP participants (*n* = 94).

	MAN-GO (versus SOEP)	Year	MAN-GO *∗* year
*Work stress*			
*E*-*R* ratio	−0.23 (−0.34, − 0.11)^ *∗∗∗*^	0.04 (0.01, 0.06)^*∗∗*^	−0.05 (−0.07, − 0.02)^*∗∗∗*^
(i) Effort	−1.26 (−2.06, − 0.45)^*∗∗*^	0.26 (0.08, 0.44)^*∗∗*^	−0.35 (−0.54, − 0.15)^*∗∗∗*^
(ii) Reward	3.64 (1.95, 5.32)^*∗∗∗*^	−0.57 (−0.91, − 0.24)^*∗∗∗*^	0.83 (0.44, 1.22)^*∗∗∗*^
Overcommitment	−1.62 (−2.66, − 0.59)^*∗∗*^	0.25 (0.03, 0.46)^*∗*^	−0.30 (−0.55, − 0.04)^*∗*^
*Mental health*			
Depressive symptoms	−4.67 (−6.90, − 2.44)^*∗∗∗*^	0.88 (0.46, 1.29)^*∗∗∗*^	−0.83 (−1.34, − 0.32)^*∗∗*^

Regression coefficients and 95% confidence intervals (CIs); mixed regression, ^*∗*^*p* < 0.05, ^*∗∗*^*p* < 0.01, and ^*∗∗∗*^*p* < 0.001.

**Table 4:** Adjusted longitudinal tracking of changes in work stress and depressive symptoms across 2006–2015 between MAN-GO participants (*n* = 94) and SOEP participants (*n* = 94).

	MAN-GO (versus SOEP)	Year	MAN-GO *∗* year
*Work stress*			
*E*-*R* ratio	−0.23 (−0.34, − 0.11)^*∗∗∗*^	0.04 (0.01, 0.06)^*∗∗*^	−0.05 (−0.07, − 0.02)^*∗∗∗*^
(i) Effort	−1.25 (−2.06, − 0.44)^*∗∗*^	0.26 (0.08, 0.44)^*∗∗*^	−0.35 (−0.54, − 0.15)^*∗∗∗*^
(ii) Reward	3.75 (2.06, 5.45)^*∗∗∗*^	−0.57 (−0.91, − 0.24)^*∗∗∗*^	0.83 (0.44, 1.21)^*∗∗∗*^
Overcommitment	−1.62 (−2.67, − 0.58)^*∗∗*^	0.25 (0.04, 0.46)^*∗*^	−0.30 (−0.55, − 0.04)^*∗*^
*Mental health*			
Depressive symptoms	−4.82 (−7.06, − 2.59)^*∗∗∗*^	0.88 (0.46, 1.29)^*∗∗∗*^	−0.83 (−1.34, − 0.32)^*∗∗*^

Regression coefficients and 95% confidence intervals (CIs); mixed regression, adjustment for age, partnership, and education at baseline, ^*∗*^*p* < 0.05, ^*∗∗*^*p* < 0.01, and ^*∗∗∗*^*p* < 0.001.

## References

[1] Berkman L., Kawachi I., Theorell T., Berkman L., Kawachi I., Glymour M. (2014). Working conditions and health. *Social Epidemiology*.

[2] (2014). *Calculating the Cost of Work-Related Stress and Psychosocial Risks*.

[3] LaMontagne A. D., Keegel T., Louie A. M., Ostry A., Landsbergis P. A. (2007). A systematic review of the job-stress intervention evaluation literature, 1990–2005. *International Journal of Occupational Medicine and Environmental Health*.

[4] Semmer N. K. (2006). Job stress interventions and the organization of work. *Scandinavian Journal of Work, Environment & Health*.

[5] Montano D., Hoven H., Siegrist J. (2014). Effects of organisational-level interventions at work on employees' health: A systematic review. *BMC Public Health*.

[6] Richardson K. M., Rothstein H. R. (2008). Effects of occupational stress management intervention programs: a meta-analysis. *Journal of Occupational Health Psychology*.

[7] Tetrick L. E., Winslow C. J. (2015). Workplace stress management interventions and health promotion. *Annual Review of Organizational Psychology and Organizational Behavior*.

[8] (2014). *2014 work and well-being survey*.

[9] (2015). *First findings: Sixth European Working Conditions Survey*.

[10] Siegrist J. (1996). Adverse health effects of high-effort/low-reward conditions. *Journal of Occupational Health Psychology*.

[11] Siegrist J., Li J. (2016). Associations of extrinsic and intrinsic components of work stress with health: a systematic review of evidence on the effort-reward imbalance model. *International Journal of Environmental Research and Public Health*.

[12] Mino Y., Babazono A., Tsuda T., Yasuda N. (2006). Can stress management at the workplace prevent depression? A randomized controlled trial. *Psychotherapy and Psychosomatics*.

[13] Aust B., Peter R., Siegrist J. (1997). Stress management in bus drivers: A pilot study based on the model of effort-reward imbalance. *International Journal of Stress Management*.

[14] Unterbrink T., Pfeifer R., Krippeit L. (2012). Burnout and effort-reward imbalance improvement for teachers by a manual-based group program. *International Archives of Occupational and Environmental Health*.

[15] Limm H., Gündel H., Heinmüller M. (2011). Stress management interventions in the workplace improve stress reactivity: a randomised controlled trial. *Occupational and Environmental Medicine*.

[16] Ruwaard J., Lange A., Bouwman M., Broeksteeg J., Schrieken B. (2007). E-mailed standardized cognitive behavioural treatment of work-related stress: A randomized controlled trial. *Cognitive Behaviour Therapy*.

[17] Bhui K. S., Dinos S., Stansfeld S. A., White P. D. (2012). A synthesis of the evidence for managing stress at work: A review of the reviews reporting on anxiety, depression, and absenteeism. *Journal of Environmental and Public Health*.

[18] Brettschneider C., Djadran H., Härter M., Löwe B., Riedel-Heller S., König H.-H. (2015). Cost-utility analyses of cognitive-behavioural therapy of depression: A systematic review. *Psychotherapy and Psychosomatics*.

[19] Leichsenring F., Leweke F., Klein S., Steinert C. (2015). The empirical status of psychodynamic psychotherapy - An update: Bambi's alive and kicking. *Psychotherapy and Psychosomatics*.

[20] Knekt P., Virtala E., Härkänen T., Vaarama M., Lehtonen J., Lindfors O. (2016). The outcome of short- and long-term psychotherapy 10 years after start of treatment. *Psychological Medicine*.

[21] Stagl J. M., Bouchard L. C., Lechner S. C. (2015). Long-term psychological benefits of cognitive-behavioral stress management for women with breast cancer: 11-year follow-up of a randomized controlled trial. *Cancer*.

[22] Karyotaki E., Smit Y., de Beurs D. P. (2016). The long-term efficacy of acute-phase psychotherapy for depression: a meta-analysis of randomized trials. *Anxiety and Depression*.

[23] Li J., Riedel N., Barrech A. (2017). Nine-year longitudinal psychosocial and mental outcomes of a stress management intervention at work using psychotherapeutic principles. *Psychotherapy and Psychosomatics*.

[24] Wagner G. G., Frick J. R., Schupp J. (2007). The German socio-economic panel study (SOEP): scope, evolution and enhancements. *Schmollers Jahrbuch: Journal of Applied Social Science Studies*.

[25] Siedler T., Schupp J., Spiess C. K. (2009). The German socio-economic panel as reference data set. *Schmollers Jahrbuch: Journal of Applied Social Science Studies*.

[26] Grosse Holforth M., Reubi I., Ruckstuhl L., Berking M., Grawe K. (2004). The value of treatment-goal themes for treatment planning and outcome evaluation of psychiatric inpatients. *International Journal of Social Psychiatry*.

[27] Strauß B. (2016). Principles of Psychodynamic Group Psychotherapy. *PPmP Psychotherapie Psychosomatik Medizinische Psychologie*.

[28] Siu A. L. (2016). Screening for depression in adults: US Preventive Services Task Force Recommendation Statement. *Journal of the American Medical Association*.

[29] Siegrist J., Wege N., Pühlhofer F., Wahrendorf M. (2009). A short generic measure of work stress in the era of globalization: effort-reward imbalance. *International Archives of Occupational and Environmental Health*.

[30] Zigmond A. S., Snaith R. P. (1983). The hospital anxiety and depression scale. *Acta Psychiatrica Scandinavica*.

[31] Vilagut G., Forero C. G., Pinto-Meza A. (2013). The mental component of the short-form 12 health survey (SF-12) as a measure of depressive disorders in the general population: Results with three alternative scoring methods. *Value in Health*.

[32] Wahl I., Löwe B., Bjorner J. B. (2014). Standardization of depression measurement: A common metric was developed for 11 self-report depression measures. *Journal of Clinical Epidemiology*.

[33] Hinz A., Brähler E. (2011). Normative values for the hospital anxiety and depression scale (hads) in the general German population. *Journal of Psychosomatic Research*.

[34] Andersen H. H., Mühlbacher A., Nübling M. (2007). Computation of standard values for physical and mental health scale scores using the SOEP version of SF-12v2. *Schmollers Jahrbuch: Journal of Applied Social Science Studies*.

[35] Herrmann C. (1997). International experiences with the hospital anxiety and depression scale: a review of validation data and clinical results. *Journal of Psychosomatic Research*.

[36] Cameron I. M., Reid I. C., Lawton K. (2010). PHQ-9: Sensitivity to change over time. *British Journal of General Practice*.

[37] Jenkinson C., Layte R., Jenkinson D. (1997). A shorter form health survey: can the SF-12 replicate results from the SF-36 in longitudinal studies?. *Journal of Public Health Medicine*.

[38] Hunger M., Holle R., Meisinger C., Rathmann W., Peters A., Schunk M. (2014). Longitudinal changes in health-related quality of life in normal glucose tolerance, prediabetes and type 2 diabetes: results from the KORA S4/F4 cohort study. *Quality of Life Research*.

[39] Vangeneugden T., Laenen A., Geys H., Renard D., Molenberghs G. (2004). Applying linear mixed models to estimate reliability in clinical trial data with repeated measurements. *Controlled Clinical Trials*.

[40] Unterbrink T., Zimmermann L., Pfeifer R. (2010). Improvement in school teachers' mental health by a manual-based psychological group program. *Psychotherapy and Psychosomatics*.

[41] Unterbrink T., Pfeifer R., Krippeit L. (2014). A manual-based group program to improve mental health: What kind of teachers are interested and who stands to benefit from this program?. *International Archives of Occupational and Environmental Health*.

[42] Moir S. (2005). Ideological influences on participatory research in occupational health and safety: a review of the literature. *New Solutions*.

[43] Hahn V. C., Binnewies C., Sonnentag S., Mojza E. J. (2011). Learning How To Recover From Job Stress: Effects of a Recovery Training Program on Recovery, Recovery-Related Self-Efficacy, and Well-Being. *Journal of Occupational Health Psychology*.

[44] Ebert D. D., Thiart H., Laferton J. A. C. (2015). Restoring depleted resources: Efficacy and mechanisms of change of an internet-based unguided recovery training for better sleep and psychological detachment from work. *Health Psychology*.

[45] Dragano N., Siegrist J., Wahrendorf M. (2011). Welfare regimes, labour policies and unhealthy psychosocial working conditions: A comparative study with 9917 older employees from 12 European countries. *Journal of Epidemiology and Community Health*.

[46] Hofmann M., Hölzel L. P., Frank F. (2015). Risk assessment for mental stress at work - A comparison within Europe. *Occupational, Social & Environmental Medicine ASU International*.

[47] Uutela A. (2010). Economic crisis and mental health. *Current Opinion in Psychiatry*.

[48] Glonti K., Gordeev V. S., Goryakin Y. (2015). A systematic review on health resilience to economic crises. *PLoS ONE*.

[49] Campos-Serna J., Ronda-Pérez E., Artazcoz L., Moen B. E., Benavides F. G. (2013). Gender inequalities in occupational health related to the unequal distribution of working and employment conditions: A systematic review. *International Journal for Equity in Health*.

[50] Semmer N., Quick and J. C., Tetrick L. E. (2011). Job stress interventions and organization of work. *Handbook of Occupational Health Psychology*.

[51] Bond F. W., Bunce D. (2001). Job control mediates change in a work reorganization intervention for stress reduction. *Journal of Occupational Health Psychology*.

[52] Hasson H., Brisson C., Guérin S. (2014). An organizational-level occupational health intervention: Employee perceptions of exposure to changes, and psychosocial outcomes. *Work & Stress*.

[53] Biron C., Ivers H., Brun J.-P. (2016). Capturing the Active Ingredients of Multicomponent Participatory Organizational Stress Interventions Using an Adapted Study Design. *Stress and Health*.

[54] Cox T., Taris T. W., Nielsen K. (2010). Organizational interventions: Issues and challenges. *Work & Stress*.

[55] Biron C., Karanika-Murray M., Cooper C. L. (2012). *Improving organizational interventions for stress and well-being: Addressing process and context*.

[56] Nielsen K., Randall R. (2013). Opening the black box: presenting a model for evaluating organizational-level interventions. *European Journal of Work and Organizational Psychology*.

[57] Karanika-Murray M., Biron C., Saksvik P. Ø. (2016). Organizational Health Interventions: Advances in Evaluation Methodology. *Stress and Health*.

[58] Gilbert-Ouimet M., Brisson C., Vézina M. (2011). Intervention study on psychosocial work factors and mental health and musculoskeletal outcomes. *HealthcarePapers*.

[59] Bourbonnais R., Brisson C., Vézina M. (2011). Long-term effects of an intervention on psychosocial work factors among healthcare professionals in a hospital setting. *Occupational and Environmental Medicine*.

