# Supplementary material for: Long-Term Effectiveness of a Stress Management Intervention at Work: A 9-Year Follow-Up Study Based on a Randomized Wait-List Controlled Trial in Male Managers
Source: Biomed Res Int. 2017 Oct 18;2017:2853813. doi: 10.1155/2017/2853813 (PMC5664277; doi:10.1155/2017/2853813)
Supplement: Supplementary file 1 — Supplementary Table 1 is for drop out analysis among the MAN-GO participants. Supplementary Table 2 is for drop out analysis among the SOEP participants. [file 2853813.f1.docx]

Supplementary Table 1: Drop out analysis: Comparison of socio-demographic characteristics as well as work stress and depressive symptoms measured at pre-intervention (2006) between those who are still present (n=94) and those who have dropped out (n=80) among the MAN-GO participants

| **Variable** | **Still present** | **Dropped out** | ***p* for difference** |
| --- | --- | --- | --- |
| ***Socio-demography*** |  |  |  |
| Mean age | 40.60 ± 6.58 | 41.26 ± 9.02 | 0.58 |
| Partner (vs. no partner) | 86 (91.49 %) | 69 (86.25 %) | 0.27 |
| Low education (vs. medium or high) | 55 (58.51 %) | 44 (55.00 %) | 0.64 |
| ***Work stress*** |  |  |  |
| E-R ratio | 0.74 ± 0.23 | 0.76 ± 0.36 | 0.61 |
| - Effort | 8.67 ± 1.78 | 8.48 ± 2.07 | 0.50 |
| - Reward | 28.65 ±4.93 | 28.31 ± 5.91 | 0.68 |
| Over-commitment | 13.98 ± 3.64 | 14.89 ± 3.40 | 0.11 |
| ***Mental health*** |  |  |  |
| Depressive symptoms | 48.66 ± 8.21 | 47.36 ± 8.10 | 0.30 |

Means and SDs for continuous variables and absolute numbers and per cent for categorical variables;

Differences were examined by t-test for continuous variables and Chi-square test for categorical variables.

Supplementary Table 2: Drop out analysis: Comparison of socio-demographic characteristics as well as work stress and depressive symptoms measured at pre-intervention (2006) between those who are still present (n=94) and those who have dropped out (n=170) among the SOEP participants

| **Variable** | **Still present** | **Dropped out** | ***p* for difference** |
| --- | --- | --- | --- |
| ***Socio-demography*** |  |  |  |
| Mean age | 41.60 ± 7.44 | 41.85 ± 7.14 | 0.78 |
| Partner (vs. no partner) | 78 (82.98 %) | 128 (24.71 %) | 0.15 |
| Low education (vs. medium or high) | 52 (55.32 %) | 97 (57.06 %) | 0.78 |
| ***Work stress*** |  |  |  |
| E-R ratio | 0.75 ± 0.40 | 0.74 ± 0.38 | 0.82 |
| - Effort | 8.39 ± 3.12 | 8.21 ± 2.78 | 0.63 |
| - Reward | 28.62 ± 5.74 | 28.43 ± 5.64 | 0.80 |
| Over-commitment | 14.17 ± 3.21 | 13.81 ± 3.51 | 0.41 |
| ***Mental health*** |  |  |  |
| Depressive symptoms | 48.86 ± 7.82 | 48.70 ± 9.09 | 0.89 |

Means and SDs for continuous variables and absolute numbers and per cent for categorical variables;

Differences were examined by t-test for continuous variables and Chi-square test for categorical variables.
